# Triangular posterior pericardiectomy and postoperative outcomes after coronary artery bypass grafting

**DOI:** 10.3389/fcvm.2026.1850675

**Published:** 2026-06-25

**Authors:** Dawid Imiełowski, Piotr Feusette, Andrzej Tukiendorf, Marek Cisowski

**Affiliations:** 1 Institute of Medical Sciences, University of Opole, Opole, Poland; 2 Faculty of Physical Education and Physiotherapy, Opole University of Technology, Opole, Poland

**Keywords:** coronary artery bypass grafting, triangular posterior pericardiectomy, taxonomy, phenotypes, C-reactive protein, pericardial effusion and drainage, postoperative atrial fibrillation

## Abstract

**Introduction:**

Postoperative atrial fibrillation (POAF) and pericardial effusion remain common complications after coronary artery bypass grafting (CABG).

**Methods:**

Posterior pericardiotomy has been proposed to reduce these events, but long-term data remain limited. We evaluated the impact of triangular posterior pericardiectomy (TPP) on early and 12-month outcomes after CABG using unsupervised taxonomic analysis of postoperative responses. This retrospective study included 489 patients undergoing CABG with cardiopulmonary bypass between 2017 and 2023. Patients underwent standard surgery (no pericardiectomy, *n* = 347) or triangular posterior pericardiectomy (*n* = 142). Unsupervised taxonomic classification based on a pentad of clinical responses–CRP dynamics (day 4-day 1 difference), pericardial fluid on day 6, postoperative drainage volume, POAF, and atrial fibrillation during 12-month follow-up–was used to identify phenotypes. Comparative analyses were performed using statistical tests.

**Results:**

Four phenotypes were identified. Phenotype 4 (*n* = 50), with the highest prevalence of TPP (92%), showed the lowest inflammatory response, absence of pericardial effusion, the lowest drainage volume (≈950 mL), and no POAF or atrial fibrillation during 12–month follow–up. Phenotypes with lower TPP prevalence showed higher CRP levels, greater drainage volumes (≈1200 mL), presence of pericardial effusion, and increased POAF incidence (cohort incidence 35%). TPP was not associated with increased pleural complications.

**Discussion:**

Unsupervised phenotypic analysis suggests triangular posterior pericardiectomy is associated with a favorable postoperative profile characterized by reduced inflammation, minimal pericardial effusion, lower drainage, and absence of early and late atrial fibrillation after CABG.

## 1 Introduction

Surgical coronary revascularization in the form of coronary artery bypass grafting (CABG) is a fundamental treatment strategy for advanced coronary artery disease, restoring blood flow to ischemic myocardium and reducing the risk of complications such as myocardial infarction and heart failure. Although effective, CABG is associated with complications occurring in the perioperative period and after discharge (1). Perioperative mortality remains low, with rates of approximately 1%–3% in most centers (2). However, risk increases in elderly patients and in those with diabetes, chronic renal or pulmonary disease, prior myocardial infarction, or advanced coronary artery disease. Multicenter studies indicate that mortality after CABG depends on patient population and may reach 7%–10% in high-risk clinical groups (3).

In the perioperative period and during follow-up after CABG, patients may develop an inflammatory response associated with increased C-reactive protein (CRP) levels. Excessive inflammation resulting from surgical trauma may lead to accumulation of inflammatory fluid in the pericardial sac, increasing cardiac compression and the risk of cardiac tamponade and postoperative atrial fibrillation (POAF) (4). In cases of excessive fluid accumulation, drainage is required to reduce the risk of severe perioperative complications, including neurological events leading to central nervous system injury or death (5).

One of the most common complications after CABG is postoperative atrial fibrillation (POAF). The incidence of atrial fibrillation after cardiac surgery ranges from 17% to 33% (6–8), and after CABG occurs in approximately 20%–40% of patients (8, 9). The mechanisms underlying POAF remain incompletely understood, but include inflammatory response, oxidative stress, autonomic dysfunction, and myocardial remodeling (10). Pharmacological prevention of POAF, including antiarrhythmic drugs, has been investigated; however, their efficacy remains uncertain and their use is limited by adverse effects (11).

An important strategy for reducing postoperative atrial fibrillation involves modification of CABG using prophylactic pericardiectomy (PP). The association between pericardial effusion and supraventricular arrhythmias was demonstrated by Mulay et al. (12), who described posterior pericardiotomy consisting of a longitudinal pericardial incision made posterior and parallel to the phrenic nerve, extending from the left inferior pulmonary vein to the diaphragm, with drains placed in the left pleural cavity and anterior mediastinum.

Although studies have shown that prophylactic pericardiectomy reduces postoperative atrial fibrillation and hospitalization-related costs (12–15), evidence regarding its effect after CABG remains inconsistent (16, 17). Differences in perioperative management, including lack of routine preoperative *β*-blocker therapy in some studies, may partly explain these discrepancies (16). In addition, data on long-term outcomes related to pericardial or pleural fluid accumulation and on modifications of the technique described by Mulay et al. (12) remain limited. Our observations suggested that modification of PP into triangular pericardiectomy (TPP) may improve effectiveness in reducing CABG-related complications, including POAF and pericardial effusion.

## 2 Aim of study

The aim of this study was to evaluate the effectiveness of triangular posterior pericardiectomy in reducing postoperative complications in patients undergoing CABG with cardiopulmonary bypass, including perioperative outcomes and one-year follow-up with assessment of risk factors.

## 3 Material and methods

This was a retrospective, non-randomized, single-center observational study. Patients with multivessel coronary artery disease and significant coronary lesions, defined according to the ESC/EACTS 2018 guidelines (18), treated at the Department of Cardiac Surgery, University Clinical Hospital in Opole between January 1, 2017, and December 31, 2023, were included. Medical records of 489 consecutive patients undergoing CABG with cardiopulmonary bypass were analyzed, including patients treated without pericardiectomy (standard approach, *n* = 347) and those who underwent modified posterior pericardiectomy in the form of triangular pericardiectomy (*n* = 142). Baseline clinical characteristics of both groups are presented in Table 1.

**Table 1:** Baseline clinical characteristics of patients according to surgical approach.

Variable	Group without TPP	Group with TPP	
	Mean ± st. dev.; median; range	Mean ± st. dev.; median; range	*p*-value
*n*=	347	142	
Age at surgery [years]	65.4 ± 7.6; 66.0; 41.0–83.0	64.1 ± 8.7; 65.0; 37.0–78.0	0.315
Ejection fraction by ECHO [%]	50.6 ± 10.8; 54.0; 5.0–70.0	50.5 ± 10.2; 53.5; 16.0–68.0	0.833
BMI	29.3 ± 4.6; 29.1; 18.1–45.2	29.1 ± 4.3; 29.0; 20.6–43.0	0.642
Total extracorporeal circulation time [min]	73.5 ± 26.2; 68.0; 30.0–200.0	78.6 ± 21.6; 81.5; 25.0–165.0	0.005
Aortic cross-clamp time [min]	44.7 ± 17.2; 41.0; 11.0–120.0	49.1 ± 13.9; 49.0; 20.0–116.0	<0.001
EuroSCORE	2.0 ± 2.0; 1.4; 0.5–16.1	2.5 ± 2.0; 1.8; 0.6–14.8	<0.001
Sex	Males: 287 (82.7%); Females: 60 (17.3%)	Males: 117 (82.4%); Females: 25 (17.6%)	0.934
CCS Class—I (1), II (2), III (3), IV (4), Acute Coronary Syndrome (5)	1: 61 (17.6%); 2: 151 (43.5%); 3: 110 (31.7%); 4: 17 (4.9%); 5: 8 (2.3%)	1: 31 (21.8%); 2: 70 (49.3%); 3: 35 (24.6%); 4: 5 (3.5%); 5: 1 (0.7%)	0.035
NYHA—without circulatory failure (0), I (1), II (2), III (3), IV (4)	0: 2 (0.6%); 1: 258 (74.4%); 2: 79 (22.8%); 3: 7 (2.0%); 4: 1 (0.3%)	0: 1 (0.7%); 1: 97 (68.3%); 2: 37 (26.1%); 3: 6 (4.2%); 4: 1 (0.7%)	0.159
Smoking—never smoked (0), former smoker (1), current smoker (2)	0: 142 (40.9%); 1: 137 (39.5%); 2: 68 (19.6%)	0: 52 (36.6%); 1: 50 (35.2%); 2: 40 (28.2%)	0.113
Hypertension	No: 20 (5.8%); Yes: 327 (94.2%)	No: 2 (1.4%); Yes: 140 (98.6%)	0.035
Hyperlipidemia	No: 9 (2.6%); Yes: 338 (97.4%)	No: 6 (4.2%); Yes: 136 (95.8%)	0.343
Lung disease	A: 293 (84.4%); B: 10 (2.9%); C: 29 (8.4%); D: 15 (4.3%)	A: 112 (78.9%); B: 4 (2.8%); C: 14 (9.9%); D: 12 (8.5%)	0.112
Recent myocardial infarction	No: 184 (53.0%); Yes: 163 (47.0%)	No: 68 (47.9%); Yes: 74 (52.1%)	0.303
Pulmonary hypertension	No: 338 (97.4%); Yes: 9 (2.6%)	No: 134 (94.4%); Yes: 8 (5.6%)	0.096

Baseline characteristics presented in Table 1 indicate that the groups were comparable with respect to age, left ventricular ejection fraction, body mass index, sex distribution, NYHA class, smoking status, hyperlipidemia, lung disease, recent myocardial infarction, and pulmonary hypertension (all *p* > .05). However, Group 2 had longer cardiopulmonary bypass and aortic cross-clamp times and higher EuroSCORE values than Group 1 (*p* = .005, *p* < .001, and *p* < .001). Differences were also observed in CCS class distribution and prevalence of hypertension (*p* = .035). Overall, although the groups were similar with respect to several baseline characteristics, differences in operative risk profile and procedural variables were present and should be considered when interpreting subsequent associations.

## 4 Brief description of surgical techniques

### 4.1 Standard surgical technique

**Figure 1:**
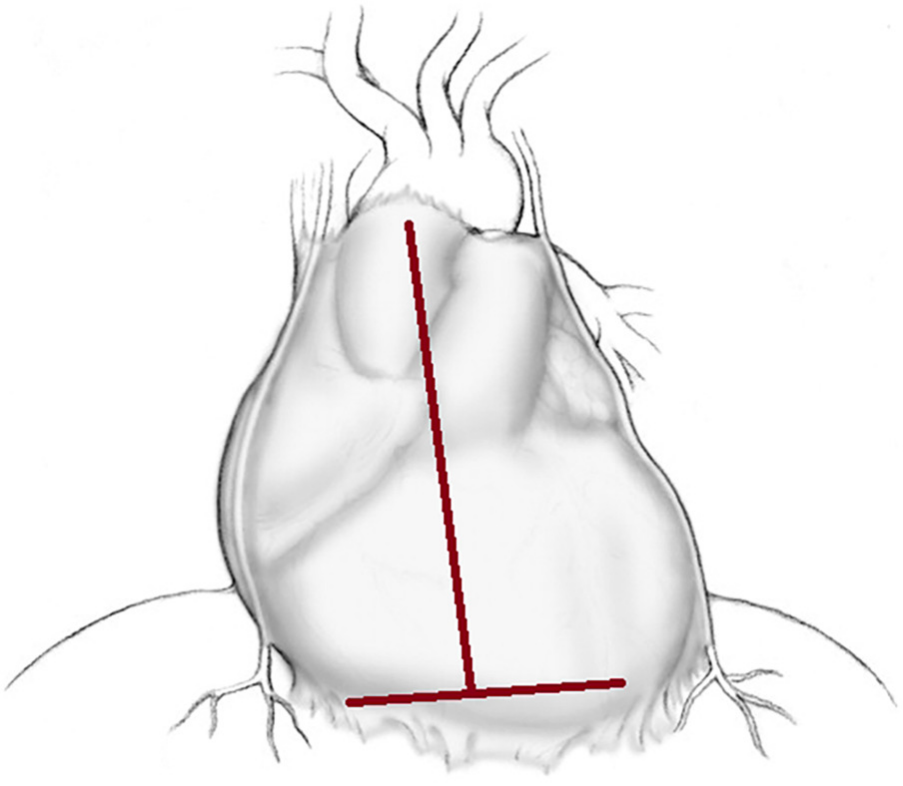
Classical T-shaped pericardial opening (original illustration by the authors). A vertical incision along the anterior heart surface combined with a transverse incision at the inferior margin of the pericardial sac forms a “T”, providing exposure of the cardiac chambers and great vessels.

Patients in the first group underwent median sternotomy with a classical T-shaped opening of the pericardial sac (Figure 1). Cardiac arrest in diastole was achieved using warm blood or cold crystalloid cardioplegia. After the procedure, the pericardium was closed anteriorly as much as possible and the chest was closed. A curved drain was placed in the left pleural cavity and a straight drain in the anterior mediastinum.

### 4.2 Modified technique—triangular posterior pericardiectomy (TPP)

**Figure 2:**
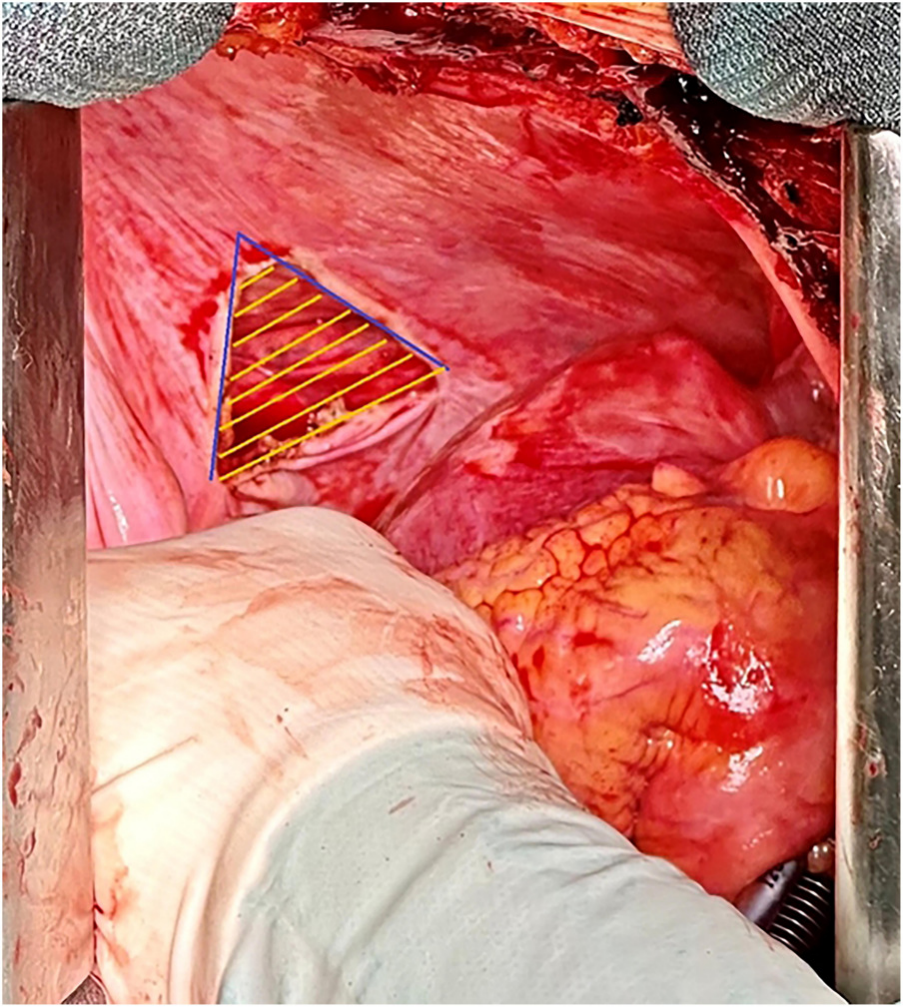
Modified triangular posterior pericardiectomy. The inverted “L” incision includes a horizontal cut below the phrenic nerve and a perpendicular incision directed toward the diaphragm, forming a triangular pericardial opening that allows the pericardial flap to fall into the left pleural cavity and facilitates drainage.

Surgery was performed via median sternotomy with a standard T-shaped pericardial opening followed by inferior posterior pericardiectomy. Unlike the technique described by Mulay et al. (12), involving a longitudinal incision parallel to the phrenic nerve, a modified approach was applied. In this technique (Figure 2), the pericardial incision was created as an inverted “L”.

A horizontal incision (≈3.5 cm) was made below and parallel to the phrenic nerve, followed by a perpendicular incision (≈3 cm) directed inferiorly above the diaphragm, forming a triangular opening (Figure 2). The pericardial flap was displaced into the left pleural cavity, improving pericardial fluid evacuation. The procedure was performed early after initiation of cardiopulmonary bypass with ventilation temporarily suspended.

## 5 General management

In all patients, myocardial arrest in diastole was achieved using warm blood or cold crystalloid cardioplegia. After the procedure, the pericardium was closed anteriorly as much as possible and the chest was closed. A curved drain was placed in the left pleural cavity and a straight drain in the anterior mediastinum.

Patients in both groups received preoperative β-blocker therapy as prophylaxis against paroxysmal atrial fibrillation. Consecutive patients undergoing CABG with cardiopulmonary bypass using the left internal mammary artery (LIMA) and the great saphenous vein (GSV) were evaluated. Anesthetic agents and surgical techniques were comparable across patients.

## 6 Postoperative management

In the cardiac surgical intensive care unit, negative pressure suction (15 cm H₂O) was applied for drainage. Mediastinal and pleural drains were removed when drainage volume was ≤100 mL over 12 h. Continuous electrocardiographic monitoring was performed during the first seven postoperative days.

Atrial fibrillation (AF) was defined by irregular R–R intervals, absence of distinct P waves on ECG, and atrial cycle length typically <200 ms (16). Postoperative atrial fibrillation (POAF) was diagnosed when an AF episode lasted longer than 30 s (17).

After AF diagnosis, treatment included *β*-blockers, calcium channel blockers, or intravenous amiodarone. In cases of hemodynamic instability, electrical cardioversion was considered. If AF persisted longer than 1 h, anticoagulation was initiated using low-molecular-weight heparin or a non-vitamin K oral anticoagulant.

Two-dimensional echocardiography was performed on postoperative day 6 to assess pericardial effusion and cardiac function. Serum potassium levels were monitored up to postoperative day 6, with supplementation to maintain levels between 4 and 5 mmol/L. CRP levels were determined on postoperative days 1 and 4.

Routine electrocardiographic monitoring was performed in outpatient cardiology clinics and during scheduled cardiac surgery outpatient visits at 2 weeks and 12 months after discharge to detect atrial fibrillation episodes. Additionally, supplementary telephone follow-up during the 12-month observation period was conducted to assess documented arrhythmic events and the need for left pleural puncture.

## 7 Statistical methods

The statistical analysis was based on unsupervised structural analysis of clinical response patterns using squared matrices of taxonomic distances between patients (symmetric dissimilarities), calculated with the Marczewski–Steinhaus (M–S) metric (19).

In this approach, the symmetric taxonomic distance (D) between two patients (A and B) is defined as:

D=|A–B|/max(A,B)

where the numerator represents the absolute difference between A and B, and the denominator their maximum value.

Unlike analyses based on a single clinical outcome, this method allows evaluation of combinations of variables within geometric frameworks, in which distances between patients are defined using a taxonomic metric. The most heterogeneous patient types identified through taxonomic analysis were used for comparative analyses with clinical risk factors using Wilcoxon rank-sum and Student's t-tests.

All statistical computations were performed using R software (20), utilizing the “cluster’ package (21) for taxonomic clustering. Data and computational procedures are available upon request.

The collected clinical material and analytical methodology were used to identify postoperative taxonomic phenotypes and evaluate their association with early and long-term clinical outcomes after CABG. The primary outcomes of the study were the occurrence of postoperative atrial fibrillation (POAF) during hospitalization and atrial fibrillation during the 12-month follow-up period, as these represented the principal clinical endpoints of interest. Secondary outcomes included postoperative inflammatory response assessed by CRP dynamics (difference between postoperative day 4 and day 1 values), presence and amount of pericardial fluid on postoperative day 6, total postoperative drainage volume, postoperative stroke, pleural complications, and other clinically relevant postoperative variables.

## 8 Results

**Figure 3:**
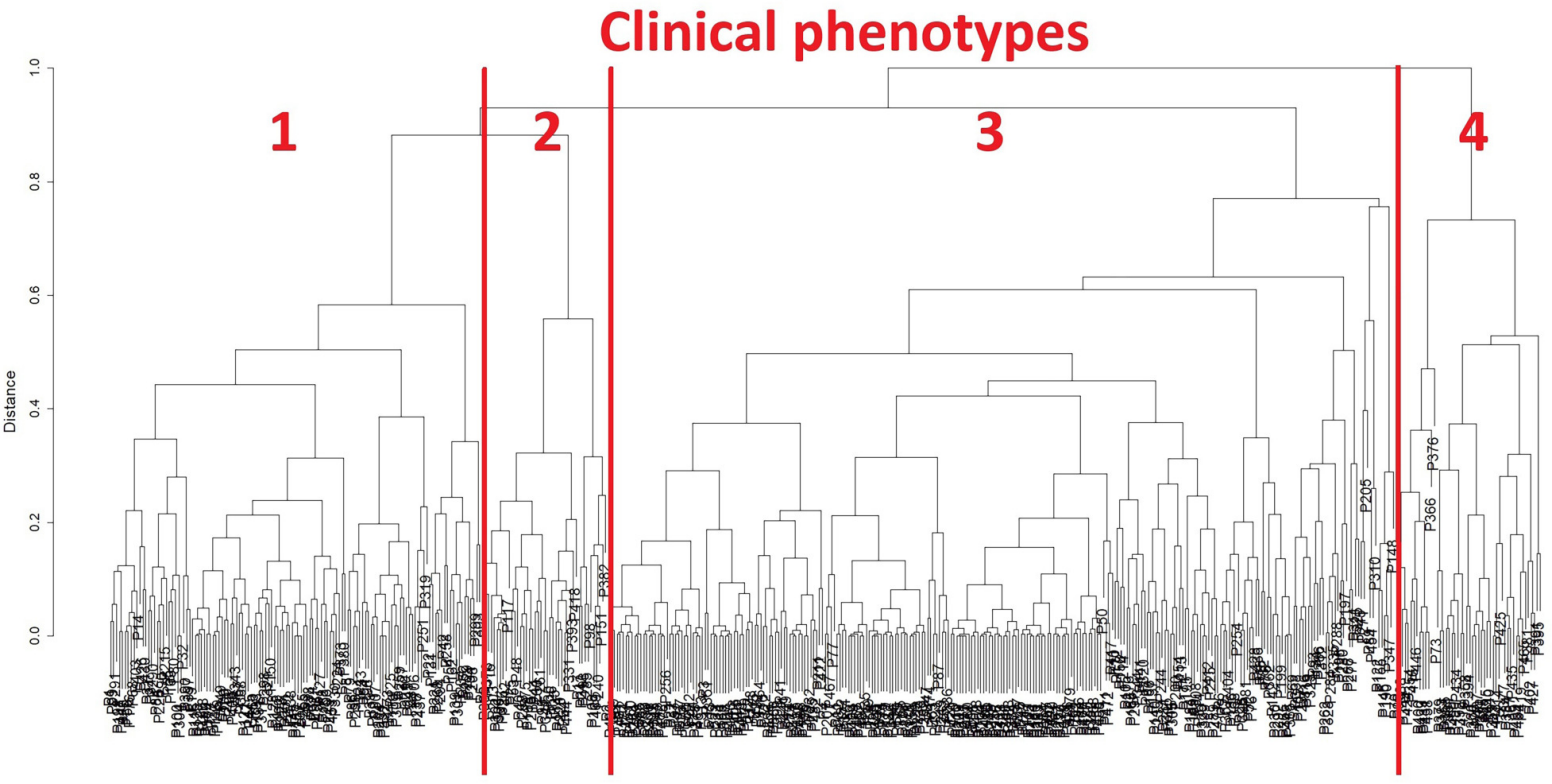
Taxonomic dendrogram based on a pentad of clinical responses identifying patient phenotypes. The dendrogram illustrates unsupervised clustering of patients into—clinical phenotypes using the following variables: difference in CRP levels between postoperative days 4 and 1, pericardial fluid on postoperative day 6, total postoperative drainage volume, postoperative atrial fibrillation, and paroxysmal atrial fibrillation within 12 months after discharge. Individual patients (“P”) are hierarchically clustered into branches represented by terminal leaves. The dendrogram should be interpreted as a visualization of similarity among postoperative response patterns, where shorter taxonomic distances indicate greater similarity between patients and larger branch separations reflect more distinct postoperative profiles. Based on the distribution of clustering distances and the corresponding scree method, the three largest thresholds identified in the dendrogram (Figure 3) were considered the most rational and clinically interpretable from the perspective of postoperative phenotypology and were therefore used to delineate four patient types—Type 1 (*n* = 127), Type 2 (*n* = 43), Type 3 (*n* = 269), and Type 4 (*n* = 50)—with boundaries indicated by vertical red lines. Descriptive statistics of the taxonomic patient phenotypes identified from the pentad of clinical responses, together with clinical risk factor characteristics showing statistically significant differences (*p* < .05), are presented in Table 2.

Among the analyzed multidimensional combinations, optimal unsupervised differentiation of patients' clinical phenotypes was achieved for a pentad of clinical responses consisting of: the difference in C-reactive protein (CRP) levels between postoperative days 4 and 1, pericardial fluid on postoperative day 6, total postoperative drainage volume, postoperative atrial fibrillation, and paroxysmal atrial fibrillation within 12 months after discharge. The taxonomic dendrogram corresponding to this analysis is presented in Figure 3.

**Table 2:** Descriptive statistics of taxonomic patient types identified from a pentad of clinical responses—including CRP difference between postoperative days 4 and 1, pericardial fluid on postoperative day 6, total postoperative drainage volume, postoperative atrial fibrillation, and paroxysmal atrial fibrillation within 12 months after discharge—together with clinical risk factor characteristics showing statistically significant differences (*p* < .05).

Variable	Phenotype 1	Phenotype 2	Phenotype 3	Phenotype 4	50
*n*=	mean ± st. dev.; median; range	127	43	269	
		mean ± st. dev.; median; range	mean ± st. dev.; median; range	mean ± st. dev.; median; range	*p*-value
CRP 1 day [mg/L]	164.19 ± 56.76; 154; 57–380	207.88 ± 47.87; 209; 57–287	157.03 ± 57.55; 147; 35–400	159.34 ± 60.94; 142; 35–323	< 0.001
CRP 4 day [mg/L]	92.24 ± 43.87; 79; 29–217	149 ± 47.53; 167; 29–213	90.59 ± 44.85; 79; 16–295	77.42 ± 40.45; 70; 16–197	< 0.001
Amount of fluid in the pericardial sac on day 6 [mL]	3.85 ± 4.45; 2; 0–21	3.21 ± 3.43; 3; 0–12	1.39 ± 3; 0; 0–16	0.04 ± 0.28; 0; 0–2	< 0.001
Total postoperative drainage [mL]	1,214.56 ± 106.49; 1,200; 850–1,650	1,227.91 ± 108.73; 1,200; 950–1,550	1,221.71 ± 85.73; 1,200; 1,050–1,700	956 ± 79.95; 950; 700–1,100	< 0.001
Age at the start of surgery [years]	67.58 ± 7.43; 68; 44–83	66.42 ± 6.97; 66; 52–79	63.89 ± 7.85; 65; 37–79	63.22 ± 8.72; 66; 39–75	< 0.001
Last preoperative serum creatinine level [mg/dL]	1.07 ± 0.34; 1; 0.53–3.04	1.20 ± 0.43; 1.08; 0.73–3.15	1.06 ± 0.31; 1; 0.46–2.95	1.14 ± 0.31; 1.10; 0.61–2.40	0.011
Total dose of amiodarone [mg]	1,861.42 ± 1,017.78; 1,800; 600–4,800	1,576.74 ± 1,456.81; 1,200; 0–5,400	0 ± 0; 0; 0–0	0 ± 0; 0; 0–0	< 0.001
Left atrial size [mm]	40.35 ± 4.73; 40; 30–54	43.86 ± 5.29; 44; 35–58	40.24 ± 4.70; 40; 29–56	41.48 ± 4.29; 41; 32–52	< 0.001
Postoperative atrial fibrillation (AF)	yes: 127 (100.0%)	no: 10 (23.3%); 1: 33 (76.7%)	no: 269 (100.0%)	no: 50 (100.0%)	< 0.001
AF episode within 12 months after discharge	no: 127 (100.0%)	yes: 43 (100.0%)	no: 269 (100.0%)	no: 50 (100.0%)	< 0.001
Preoperative renal failure—normal (0), moderate (1), severe (2), dialysis (3)	0: 59 (46.5%); 1: 61 (48.0%); 2: 7 (5.5%)	0: 16 (37.2%); 1: 21 (48.8%); 2: 6 (14.0%)	0: 114 (42.4%); 1: 139 (51.7%); 2: 14 (5.2%); 3: 2 (0.7%)	0: 5 (10.0%); 1: 41 (82.0%); 2: 3 (6.0%); 3: 1 (2.0%)	< 0.001
Postoperative stroke	no: 122 (96.1%); yes: 5 (3.9%)	no: 40 (93.0%); yes: 3 (7.0%)	no: 268 (99.6%); yes: 1 (0.4%)	no: 50 (100.0%)	0.004
Surgical group—without TPP, with TPP	without PP: 112 (88.2%); with PP: 15 (11.8%)	without PP: 37 (86.0%); with PP: 6 (14.0%)	without PP: 194 (72.1%); with PP: 75 (27.9%)	without PP: 4 (8.0%); with PP: 46 (92.0%)	< 0.001
Preoperative cardiac rythm—sinus rhythm (A), atrial fibrillation (B)	A: 102 (80.3%); B: 25 (19.7%)	A: 26 (60.5%); B: 17 (39.5%)	A: 247 (91.8%); B: 22 (8.2%)	A: 45 (90.0%); B: 5 (10.0%)	< 0.001
Cerebrovascular disease—no (A), TIA (B), ischemic stroke (C)	A: 117 (92.1%); B: 5 (3.9%); C: 5 (3.9%)	A: 35 (81.4%); B: 7 (16.3%); C: 1 (2.3%)	A: 248 (92.2%); B: 14 (5.2%); C: 7 (2.6%)	A: 49 (98.0%); B: 1 (2.0%)	0.037

The greatest differentiation between the two surgical groups—Group 1 (TPP=No) and Group 2 (TPP=Yes)—was observed in phenotype 4, in which prophylactic pericardiectomy was performed in 92% of patients (46/50). The mean age in this phenotype was 66 years and was comparable across phenotypes. Phenotype 4 was characterized by the lowest postoperative inflammatory response, with mean CRP levels of 142 mg/L on postoperative day 1 and 70 mg/L on postoperative day 4. On postoperative day 6, no pericardial effusion was detected on ultrasonography, and the mean total postoperative drainage volume was the lowest among phenotypes (950 mL). Neither early postoperative atrial fibrillation nor paroxysmal atrial fibrillation during 12-month follow-up was observed. These patients did not receive amiodarone during hospitalization. The mean left atrial appendage diameter was 41 mm, similar to phenotypes 1 and 3. No ischemic stroke occurred, although 10% had a history of paroxysmal atrial fibrillation preoperatively. Preoperative neurological burden was present in 2% of patients as transient ischemic attack (TIA). Most patients had reduced glomerular filtration rates ranging from 50 to 85 mL/min, and the mean preoperative creatinine level was 1.1 mg/dL (Table 2).

From a statistical perspective, phenotype 3 was less homogeneous than phenotype 4. Patients undergoing prophylactic pericardiectomy accounted for 27.9% of this group (75/269). The mean age was 65 years. Postoperative inflammatory response was slightly higher than in phenotype 4, with mean CRP levels of 147 mg/L on postoperative day 1 and 79 mg/L on postoperative day 4. Similarly to phenotype 4, no pericardial effusion was detected on postoperative day 6. However, total postoperative drainage volume was higher than in phenotype 4 and similar to phenotypes 1 and 2 (mean 1,200 mL). No atrial fibrillation occurred during the early postoperative period or during 12-month follow-up, and no amiodarone was administered. The mean left atrial appendage diameter was 40 mm. One patient experienced an ischemic stroke early after surgery. Preoperatively, 22 patients (8.2%) had paroxysmal atrial fibrillation. TIA occurred in 5.2% of patients before surgery and ischemic stroke in 2.6%. Renal function was preserved in 42.4% of patients, whereas 51.7% had reduced glomerular filtration rates of 50–85 mL/min, similar to phenotype 4. The mean preoperative creatinine level was 1.0 mg/dL.

Clinical phenotypes 1 and 2 comprised patients in whom prophylactic pericardiectomy (TPP) was performed least frequently, accounting for 11.8% and 14% of cases. The mean age in these subgroups was 68 and 66 years and was comparable to the remaining phenotypes. Phenotype 2 exhibited the highest postoperative inflammatory response, with mean CRP levels of 209 mg/L on postoperative day 1 and 167 mg/L on postoperative day 4. In contrast, CRP levels in phenotype 1 were similar to those observed in phenotypes 3 and 4, averaging 154 mg/L on day 1 and 79 mg/L on day 4.

In both phenotypes 1 and 2, pericardial fluid was detected on postoperative day 6, with mean effusion thickness of approximately 2 mm and 3 mm, respectively. Total postoperative drainage volume was comparable to phenotype 3 and averaged approximately 1,200 mL. Perioperative atrial fibrillation occurred in all 127 patients classified as phenotype 1 and in 33 patients (76.7%) in phenotype 2. During the 12-month follow-up period, at least one episode of atrial fibrillation was recorded in all patients with phenotype 2, whereas no such episodes were observed in phenotype 1.

The mean cumulative dose of amiodarone during hospitalization was 1,800 mg in phenotype 1 and 1,200 mg in phenotype 2. The left atrial appendage diameter was largest in phenotype 2 (≈44 mm), while in phenotype 1 it averaged 40 mm and was comparable to phenotypes 3 and 4. In the early postoperative period, isolated ischemic stroke occurred in 3.9% and 7% of patients in phenotypes 1 and 2, respectively.

**Figure 4:**
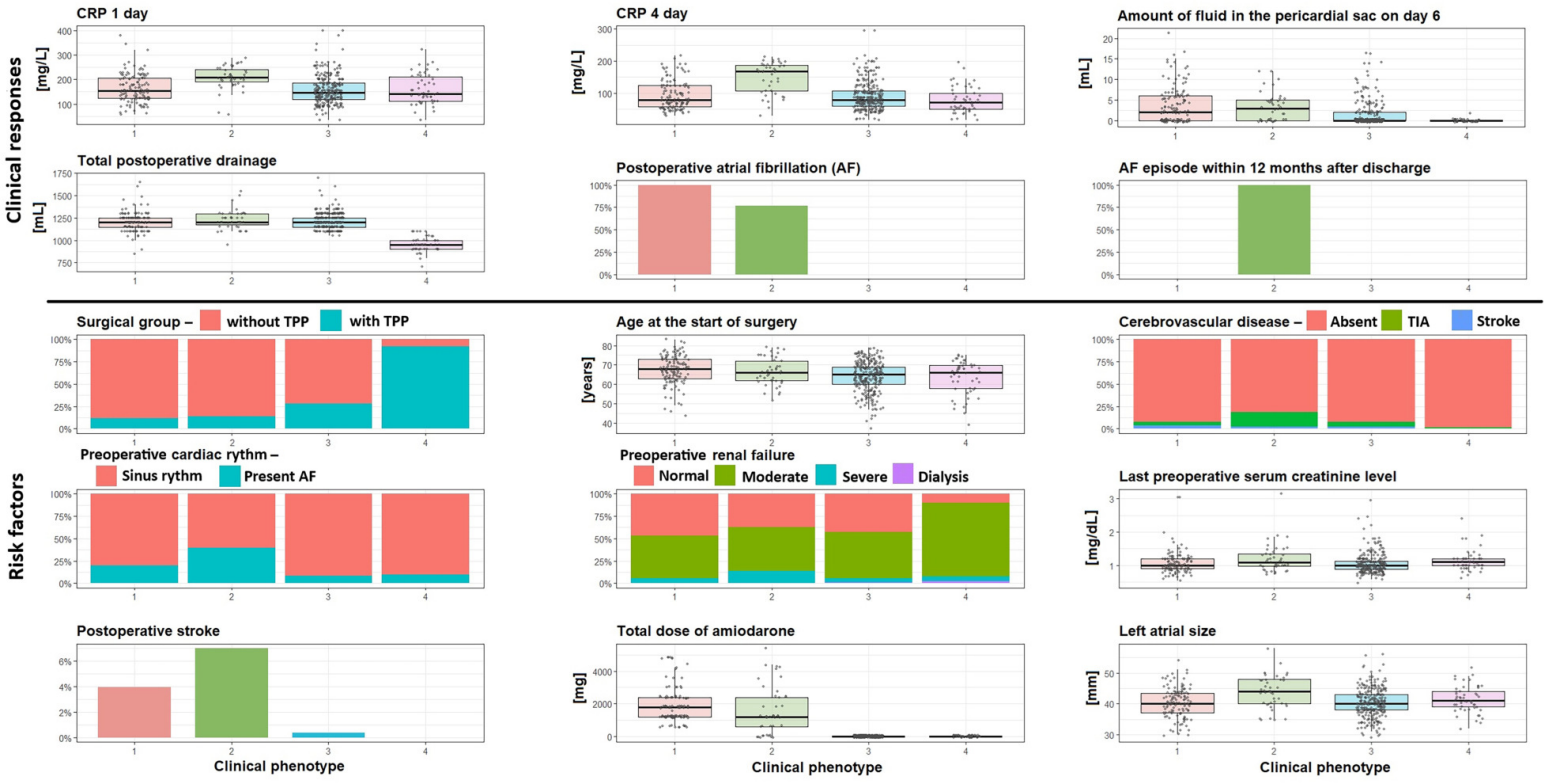
Box plots, bar charts, and stacked bar plots illustrating clinical response variables and corresponding clinical risk factor characteristics across unsupervised taxonomic phenotypes (statistically significant differences, *p* < .05).

Preoperatively, atrial fibrillation was present in 19.7% of patients in phenotype 1 and 39.5% in phenotype 2. Transient ischemic attack occurred in 3.9% and 16.3% of patients, while preoperative ischemic stroke was documented in 3.9% of phenotype 1 and 2.3% of phenotype 2. Severely impaired renal function (glomerular filtration rate <50 mL/min) was observed in 5.5% of patients in phenotype 1% and 14% in phenotype 2. Preoperative creatinine levels were comparable between the subgroups and averaged approximately 1.0 mg/dL. A graphical visualization of the results presented in Table 2 is shown in Figure 4.

The distributions of clinical response variables and risk factor profiles across the four taxonomic phenotypes are illustrated in Figure 4 as box plots, bar charts, and stacked bar plots, complementing numerical results presented in Table 2. The graphical presentation highlights distinct clinical patterns among phenotypes, particularly the favorable profile of phenotype 4, characterized by lower inflammatory response (CRP), minimal pericardial fluid accumulation, reduced postoperative drainage, and absence of postoperative and late atrial fibrillation.

## 9 Discussion

Patients after coronary artery bypass grafting (CABG) experience various complications. In the perioperative period, an increase in C-reactive protein (CRP) occurs as part of the inflammatory response to surgery. Among the four taxonomically identified clinical phenotypes, the lowest CRP level on postoperative day 4 (mean = 77 mg/L) was observed in phenotype 4, which had the highest proportion of patients (92%) undergoing modified prophylactic pericardiectomy. In contrast, CRP levels were approximately twofold higher (149 mg/L) in phenotype 2, in which prophylactic pericardiectomy was not performed in most patients (86%). These findings suggest a more favorable inflammatory profile in patients treated with triangular pericardiectomy.

Surgical group 2 (TPP=yes) also had a lower risk of cardiac tamponade and pericardial fluid accumulation, which may translate into better cardiac hemodynamics, particularly in patients with reduced ejection fraction (EF). The presence of pericardial fluid in patients with low EF leads to earlier reduction in cardiac output than in those with preserved EF (22). This may result in low cardiac output syndrome (LCOS) with manifestations such as hypotension, oliguria, neurological disorders including transient ischemic attack (TIA) and stroke, and the need for inotropic agents or mechanical circulatory support (22). In our study, pericardial fluid in phenotype 4 was minimal or absent. During postoperative follow-up, phenotypes 2, 3, and 1 showed significantly higher postoperative drainage volume (≈1,200 cm^3^) compared with phenotype 4 (≈950 cm^3^) (*p* < .05).

These favorable outcomes of triangular pericardiectomy may also relate to the technique used for harvesting the internal mammary artery, which influences surgical trauma and inflammatory response (23). Higher inflammatory markers were observed when the artery was harvested as a pedicled graft with surrounding tissues. This technique (TPP=no) is more invasive and results in a larger surgical wound compared with the skeletonized technique (TPP=Yes), in which the artery is harvested in an isolated manner.

Most importantly, prophylactic pericardiectomy (TPP) may contribute to reducing the risk of serious complications after CABG, particularly postoperative atrial fibrillation (POAF) and related central nervous system ischemic events. POAF episodes, occurring in approximately 20%–40% of patients after CABG, are associated with prolonged hospitalization, increased neurological complications, and worse long-term prognosis (24). Similarly, the study by Sahdev et al. (25) recently demonstrated that posterior pericardiotomy (PP) was associated with a reduced incidence of postoperative atrial fibrillation after coronary artery bypass grafting surgery (12.5% in the control group vs. 9.4% in the PP group; *p* = .027). In the analyzed cohort of 489 patients, POAF occurred in 169 individuals (35%), placing the incidence in the upper range of reported statistics. Therefore, the benefits of TPP emphasized in our study may have clinical relevance.

Recent reports have demonstrated an association between elevated CRP levels and episodes of atrial fibrillation. Although CRP itself is not a pathogenic factor but rather a biomarker reflecting inflammatory response, such a state may lead to myocardial cellular injury and disturbances in electrical conduction (4).

Using the applied taxonomic approach, additional clinically relevant observations can be derived. Among patients who underwent prophylactic pericardiectomy (TPP), no ischemic stroke occurred during either hospitalization or the one-year follow-up, even among those with a history of atrial fibrillation before surgery.

In summary, the applied unsupervised taxonomic classification of patients after CABG, based on a “pentad” of clinical responses (CRP dynamics, pericardial fluid on postoperative day 6, total postoperative drainage volume, postoperative atrial fibrillation, and atrial fibrillation during 12-month follow-up), enabled identification of four statistically distinct phenotypes (*p* < .05) with different postoperative risk profiles. Importantly, the benefits identified were predominantly associated with phenotype 4, characterized by the highest proportion of patients undergoing triangular posterior pericardiectomy (TPP=Yes). The most significant finding in this phenotype was the absence of postoperative atrial fibrillation and atrial fibrillation during the 12-month follow-up. The reduction in inflammatory response and pericardial effusion, resulting in lower postoperative drainage and elimination of arrhythmogenic stimuli, may contribute to prevention of POAF and indicate potential benefits of triangular posterior pericardiectomy after surgical coronary revascularization.

This hypothesis is also reflected in the analysis of phenotype 3, which was characterized by a relatively high proportion of patients undergoing prophylactic pericardiectomy, although to a lesser extent—27% received the modified procedure. Patients classified as phenotype 3 did not experience postoperative atrial fibrillation or atrial fibrillation during the 12-month follow-up, and pericardial fluid on postoperative day 6 was rare or absent. These findings suggest that the observed effect may reflect a broader impact of triangular posterior pericardiectomy as a modifier of the pericardial environment, with clinical expression dependent on baseline burden and coexisting risk factors.

By generalizing postoperative and 12-month observations, our results indicate that phenotypes characterized by low intensity of the postoperative “pentad” were rarely associated with recurrent atrial fibrillation. At the same time, the absence of differences in clinically significant pleural effusions requiring intervention indicates that triangular posterior pericardiectomy does not increase the risk of pleural complications (26, 27).

From a clinical perspective, the taxonomic approach used in this study was intended to identify naturally occurring postoperative response patterns rather than predefined risk categories. In contrast to conventional analyses focused on isolated variables, taxonomic phenotyping allows simultaneous characterization of multidimensional clinical profiles. Such an approach may contribute to postoperative risk stratification and facilitate identification of patient subgroups with different clinical trajectories after CABG. Recognition of favorable and unfavorable postoperative phenotypes may potentially support individualized monitoring and management strategies.

We acknowledge, however, the limitations of this interpretation, including the retrospective single-center design and the fact that identified phenotypes do not constitute evidence of causality. Nevertheless, the consistency of observations, their concordance with the pathophysiology of postoperative atrial fibrillation, and the association of posterior pericardiectomy with a more favorable profile across multiple dimensions of the clinical “pentad” suggest potential clinical value. Therefore, the observed relationships should be interpreted cautiously and require validation in independent prospective studies.

## 10 Conclusion

In the authors' key message derived from this study, the original application of an unsupervised taxonomic approach based on a postoperative “pentad” of clinical responses, through identification of distinct post-CABG risk phenotypes, supports the thesis that triangular posterior prophylactic pericardiectomy (TPP) was more frequently present in phenotypes characterized by a favorable clinical course. These phenotypes were marked by lower inflammatory response, absence of pericardial effusion, reduced postoperative drainage volume, and reduction in early and late atrial fibrillation.

## Data Availability

The raw data supporting the conclusions of this article will be made available by the authors, without undue reservation.
